# Polyanionic Cathode Materials for Practical Na-Ion Batteries toward High Energy Density and Long Cycle Life

**DOI:** 10.1021/acscentsci.3c00907

**Published:** 2023-09-08

**Authors:** Chunliu Xu, Junmei Zhao, Chao Yang, Yong-Sheng Hu

**Affiliations:** † CAS Key Laboratory of Green Process and Engineering, Institute of Process Engineering, Chinese Academy of Sciences, Beijing 100190, China; ⊥ Key Laboratory of Green and High-value Utilization of Salt Lake Resources, Chinese Academy of Sciences, Beijing 100190, China; § School of Chemical Engineering, University of Chinese Academy of Sciences, Beijing 100049, China; ‡ Key Laboratory for Renewable Energy, Beijing Key Laboratory for New Energy Materials and Devices, Beijing National Laboratory for Condensed Matter Physics, Institute of Physics, Chinese Academy of Sciences, Beijing, 100190, China

## Abstract

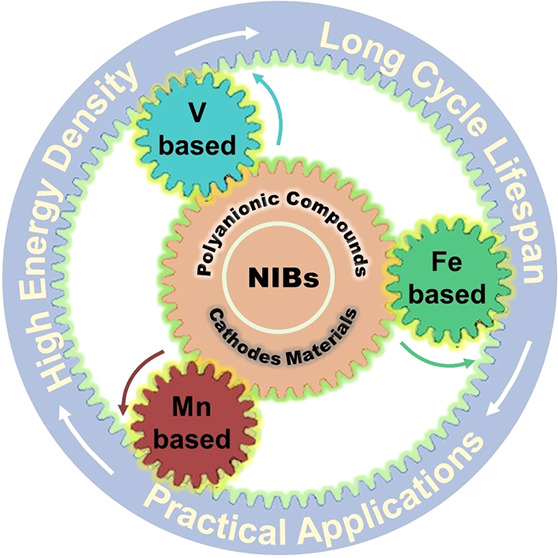

Na-ion batteries (NIBs) as a supplement to Li-ion batteries deliver huge application potential in the field of grid-scale energy storage. At present, it is a particularly imperative to advance commercialization of the NIBs after ten years of intensive research. Among the exploited cathodes for NIBs, polyanionic compounds have great commercial prospects due to their favorable ion diffusion channels, high safety, and superior structure stability determined by their unique structure framework; however, there is still a long way to go before large-scale industrialization can be realized. This outlook summarizes the recent progress of polyanion-type cathodes for NIBs and includes V-based, Fe-based, and Mn-based polyanionic compounds toward high energy density and long cycle lifespan. The remaining challenges and guidelines/suggestions for the design of the practically available polyanionic cathode materials with desirable energy density and cycling performance are presented. We hope that this outlook can provide some insights into the development of polyanionic cathodes for practical NIBs toward commercialization.

## Abstract 2

Directional guidance is given for the development of polyanionic cathodes including typical V-based, Fe-based, and Mn-based compounds with high energy density and long cycle life for Na-ion batteries toward commercialization.

## 1 Introduction

In recent decades, faced with the increasingly serious resource and environment crises, renewable energy and energy conservation/conversion are receiving great attention and market interest at a globalization level.^1−3^ Stationary secondary batteries, as one of the most promising energy storage technologies, can integrate intermittent energy sources (e.g., solar, wind, and tidal energy) into a constant and steady smart grid, allowing stable operation of the power production.^4^ Li-ion batteries (LIBs) have been used in a wide range of applications, including (plug-in hybrid) electric vehicles, mobile appliances, and energy storage systems since their first commercialization in 1991.^2^ However, the growing consumption of lithium has led to the steep rise in price due to poor reserves and the nonuniform geographic distribution of lithium resource.^4^ In contrast, sodium element is highly abundant in the Earth’s crust with a relatively even distribution, which makes Na-ion batteries (NIBs) considered as one of the most prospective alternative technologies to LIBs.^5,6^ Furthermore, thanks to the high similarities in chemical properties between Na and Li, those industrial infrastructures for manufacturing prismatic or cylindrical LIBs can be directly applied for fabricating NIBs with only a few alterations.^5,7,8^ Besides, the cheaper Al foil could be allowed as the current collector for the anodes in NIBs (Cu foil for LIBs), which could decrease the overall cost of NIBs.^1^ Despite these advantages of NIBs, the heavier atom weight (23 vs 6.94 g mol^–1^) and larger ionic radius (1.02 vs 0.67 Å) indicate a slightly lower capacity delivery and slower ion transfer kinetics of NIBs in contrast to LIBs.^9^ These characteristics finally contribute to the low energy density and unfavorable transport kinetics of NIBs. To enhance the competitive advantage of NIBs against LIBs, the further increase of the energy density and cycling life are the main development directions for the current NIBs.^1,2,7^ The comprehensive performance of the current NIBs, including voltage output, capacity delivery, cycling duration, and safety concerns are strongly dependent on the properties of electrode materials.^7^ So far, it seems that much more progress has been made in anodes than cathodes, especially for the representative hard carbon anode, which has delivered a comparable specific capacity with graphite for LIBs.^6^ However, the energy density and cycle lifespan of the cathode materials have already become the main bottlenecks for practical NIBs.

To date, Prussian blue analogues (PBAs), the layered metal oxides (TMOs), and polyanionic compounds have the most commercial prospects among the various exploited cathode materials for NIBs.^1,7^ PBAs attain wide attention and research interest due to their high theoretical capacity, 3D open crystal structure, and low cost of raw materials (e.g., Fe and Mn based Prussian blue analogues).^10^ However, the fast precipitation kinetics for synthesis of PBA cathodes usually leads to the generation of considerable coordination water and lattice vacancies, which finally deteriorate the electrochemical performance. The thereby obtained superfine grains also result in a low volumetric energy density, restricting their practical applications. The TMOs usually deliver high discharge capacity but suffer from a slope-like voltage profile within a wide potential range, rendering a relatively low average voltage (usually not higher than 3.4 V).^6,11^ On the other hand, the complex phase transitions are caused by the stacking modifications with slabs gliding in the deep de-sodiation state, which is considered as one of the decisive factors in the structural instability. The compact lattice structures also contribute to the sluggish migration kinetics of the Na^+^ charge carriers. Besides, the potential oxygen release of the TMO cathode is another safety issue of concern.^7^ Alternatively, polyanion-type compounds possess favorable ion diffusion channels, high safety, and superior structure stability determined by the unique framework of the structure. These features make polyanionic cathodes one of the most promising cathodes for NIBs.^12−14^

**Figure 1:**
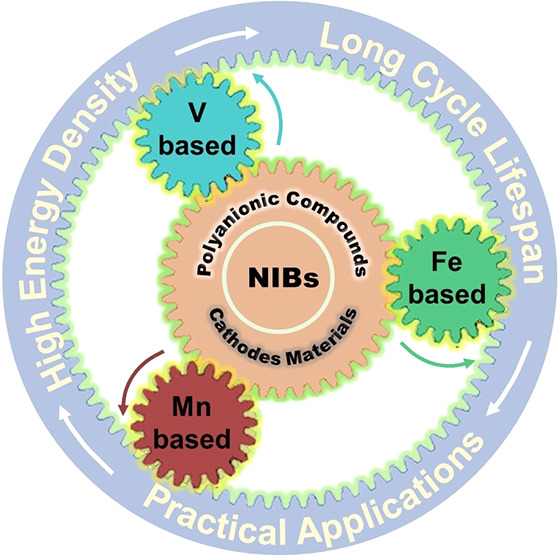
Schematic illustration of the polyanionic cathode materials for practical NIBs toward high energy density and long cycle lifespan.

In this outlook, we summarize the recent progress of typical polyanionic cathodes with high application potential for NIBs, mainly including various V-based, Fe-based, and Mn-based polyanionic compounds (Figure 1). Especially, we focus on the material design, structure characterization, reaction mechanisms, and electrochemical behavior, thus rendering an elucidation of the relationship between structure and performance. Besides, we also attempt to provide some constructive suggestions toward obtaining better electrochemical properties and insights into the research and application of polyanionic cathodes for NIBs.

## 2 Vanadium Based Polyanionic Compounds

### 2.1 NASICON Vanadium-Based Phosphates

#### Na_3_V_2_(PO_4_)_3_

**Figure 2:**
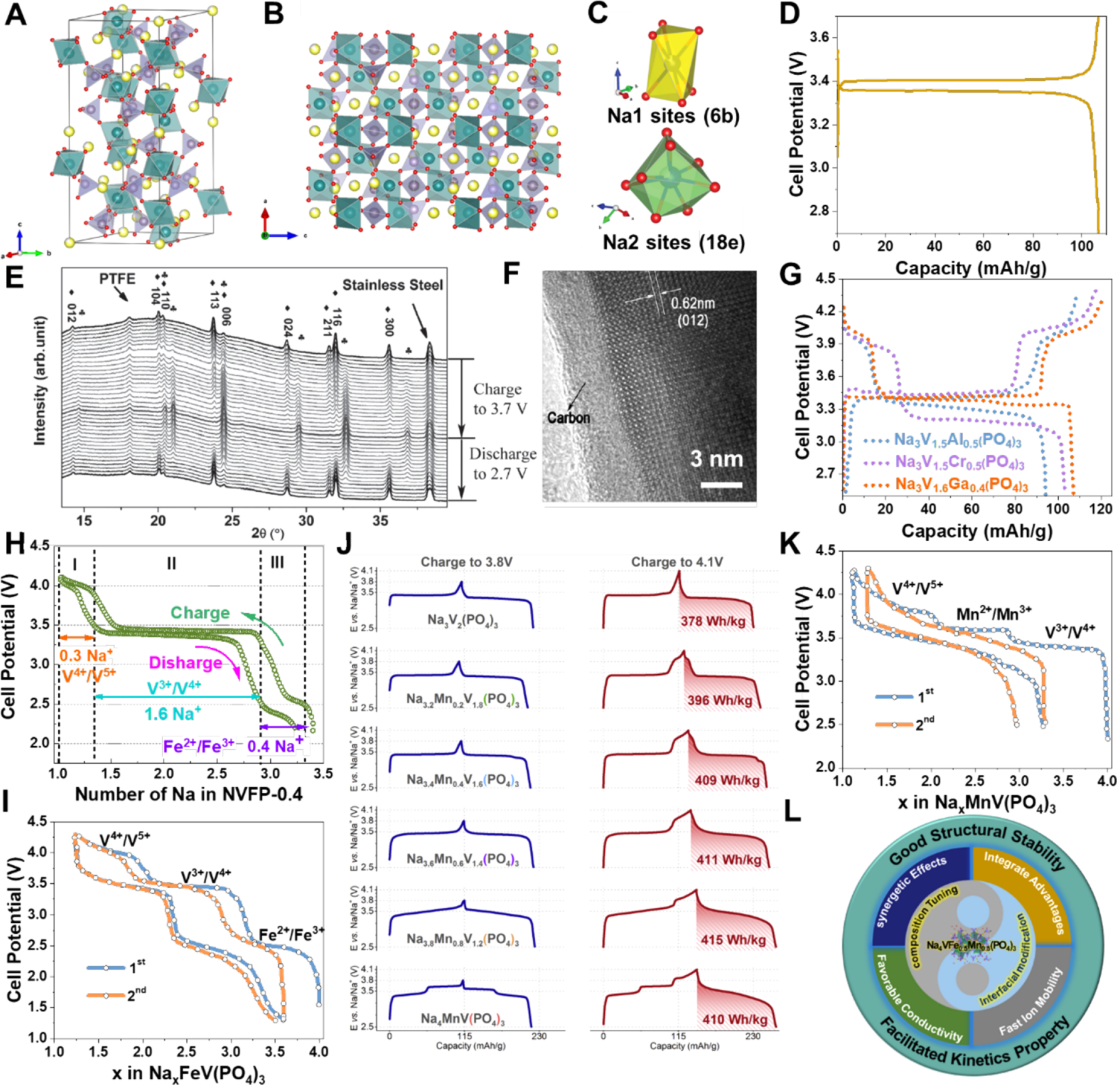
(A-B) Crystal structure of Na_3_V_2_(PO_4_)_3_ along different viewing direction. Yellow, blue, purple, and red balls represent Na, V, P and O, respectively. (C) Polyhedral structures of Na1O_6_ and Na2O_8_ site from the crystal structure of Na_3_V_2_(PO_4_)_3_, red ball is O, blue ball is Na. Reproduced with permission from ref (15). Copyright 2014 Wiley-VCH. (D) Typical charge/discharge profiles of Na_3_V_2_(PO_4_)_3_ half cells. (E) *In situ* XRD patterns of the Na_3_V_2_(PO_4_)_3_ half cells between 2.7 and 3.7 V at 0.1 C. Reproduced with permission from ref (18). Copyright 2013 Wiley-VCH. (F) HRTEM image of the Na_3_V_2_(PO_4_)_3_/C sample. Reproduced with permission from ref (16). Copyright 2012, Elsevier. (G) Charge/discharge profiles comparison between Na_3_V_1.5_Al_0.5_(PO_4_)_3_, Na_3_V_1.5_Cr_0.5_(PO_4_)_3_, and Na_3_V_1.6_Ga_0.4_(PO_4_)_3_, data from refs (21−23). (H) Charge/discharge profiles of Na_3.4_V_1.6_Fe_0.4_(PO_4_)_3_ half cells, data from ref (24). (I) Voltage-composition electrochemical profile of presodiated Na_4_FeV(PO_4_)_3_ during the first two cycles between 1.3 and 4.3 V at 0.05 C, data from ref (25). (J) Charge/discharge curves for Na_3+*x*_Mn_*x*_V_2-*x*_(PO_4_)_3_ (0 ≤ *x* ≤ 1) cycled in the 2.5–3.8 V and 2.5–4.1 V at 0.1C. Reproduced with permission from ref (26). Copyright 2020, Elsevier. (K)Voltage-composition electrochemical profile of Na_4_MnV(PO_4_)_3_ during the first two cycles between 2.5 and 4.3 V at 0.05 C. Reproduced with permission from ref (27). Copyright 2018 Wiley-VCH. (L) Schematic illustration of Na_4_Mn_0.5_Fe_0.5_V(PO_4_)_3_ with enhanced electrochemical performance. Reproduced with permission from ref (28). Copyright 2021 Wiley-VCH.

As a typical Na super-ionic conductor (NASICON)-type compound, Na_3_V_2_(PO_4_)_3_ has been widely investigated as a cathode for NIBs due to its stable framework and facilitated ion transportation.^13^ Na_3_V_2_(PO_4_)_3_ usually belongs to a rhombohedral crystal structure with an *R*3̅*c* space group, where VO_6_ octahedra and PO_4_ tetrahedra were connected in a corner-sharing way to construct a three-dimensional framework with spacious ion transfer channels (Figure 2A-B). According to different oxygen environments, Na atoms were distributed on two different crystallographic sites, i.e., 6-fold-coordination Na1 sites (6b) and 8-fold-coordination Na2 (18e) (Figure 2C).^15^ It was well recognized that only the Na^+^ located at Na2 sites could be electrochemically extracted from Na_3_V_2_(PO_4_)_3_, corresponding to a two-electron reaction through access to V^3+^/V^4+^ redox couples (∼3.4 V, hereinafter vs Na/Na^+^) (Figure 2D).^16^ This has been confirmed by the Hu’s group by the annular-bright-field scanning transmission electron microscopy (ABF-STEM) and nuclear magnetic resonance (NMR) spectroscopy technologies.^15^ It was found when Na is extracted from Na_3_V_2_(PO_4_)_3_ to form NaV_2_(PO_4_)_3_, Na^+^ located at Na2 sites is fully extracted but the rest of the Na remains at Na1 sites, indicating the electrochemical inertia of those Na^+^ residing in Na1 sites. However, this does not mean that Na^+^ in the Na1 sites is always immobile because the multidimensional Na migration could follow an ion exchange mode of Na2–Na1–Na2 during an electrochemical reaction.^17^ In practical circumstances, the real Na migration route in Na_3_V_2_(PO_4_)_3_ could be more intricate and complex, which entails more advanced in situ characterization techniques to track and capture the structural details. By in situ X-ray diffraction (XRD) techniques, Hu et al. revealed the possible biphasic reaction mechanism between Na_3_V_2_(PO_4_)_3_ and NaV_2_(PO_4_)_3_, as shown in Figure 2E, which finally contributes to a total volume change of 8.26%, close to that of LiFePO_4_ cathodes for LIBs.^18^ Such a small volume expansion/contraction of the lattice unit should be responsible for its superior structure stability. However, the poor electronic conductivity greatly limits the electrochemical performance of Na_3_V_2_(PO_4_)_3_ cathodes. In response to this, in 2012, Hu et al. first proposed the carbon coating modification on the surface of Na_3_V_2_(PO_4_)_3_ particles using sugar as the carbon source, which enables a uniform carbon layer of ∼5 nm (Figure 2F) and thereby increased reversible capacity with enhanced cycling stability.^16^ Subsequently, Yu’s group constructed a hierarchical carbon coating structure, that is, the carbon-coated nanoparticles distributed in an amorphous carbon matrix or reduced graphene-oxide framework, to improve the electrochemical properties of Na_3_V_2_(PO_4_)_3_ cathodes. Their results indicate that Na_3_V_2_(PO_4_)_3_ coated by hierarchical carbon architecture could realize a theoretical reversible capacity (∼118 mA h g^–1^) and excellent rate performance, which are even superior to those of LiCoO_2_ and LiFePO_4_ cathodes for LIBs.^19,20^ Considering the real availability and simple operation, until now, the carbon coating process has been indispensable in preparing Na_3_V_2_(PO_4_)_3_ and its analogues to reach the desired electrochemical performance.

#### Metal Doped Na_3_V_2_(PO_4_)_3._

To further increase the energy density, the extraction of the third Na^+^ from Na_3_V_2_(PO_4_)_3_ through activation of the V^4+^/V^5+^ redox couples is always expected. However, the access to extra capacity cannot be realized in Na_3_V_2_(PO_4_)_3_ even at the upper extended voltage window to 4.5 V due to the limited active Na^+^ number (Na2).^17^ Thus, Hu et al. proposed to utilize benign elements to partially replace the V in the NASICON structure, expecting to reduce the use of vanadium and attain the possibility of the appearance of new electrochemical behavior.^1^ Several representative compounds designed by the trivalent ion doping, such as Na_3_V_1.5_Al_0.5_(PO_4_)_3_,^21^ Na_3_V_1.5_Cr_0.5_(PO_4_)_3_,^22^ Na_3_V_1.5_Fe_0.5_(PO_4_)_3_,^21,29,30^ and Na_3_V_1.6_Ga_0.4_(PO_4_)_3_^23^ have been reported to afford the accessibility of V^4+^/V^5+^ (Figure 2G). However, these compounds are less attractive because the electrochemical reaction was limited to the exchange of only two electrons. Taking the Na_3_V_1.5_Al_0.5_(PO_4_)_3_ cathode for example, the oxidation of V^3+^/ V^4+^ (1.5 mol V) corresponds to 1.5 mol Na^+^ involved in the reaction, and the 0.5 mol Na^+^ could be further extracted to guarantee partial V^4+^/V^5+^ transition. The remaining 1.0 mol of Na located at Na1 sites was still electrochemically inert. In contrast, the substitution by electrochemically active divalent elements including Fe^2+^ and Mn^2+^ renders an opportunity to reach a larger reversible capacity by exceeding the two-electron reaction. Our group designed a Na-rich Na_3.4_V_1.6_Fe_0.4_(PO_4_)_3_ cathode by Fe^2+^ substitution, which enables a reversible capacity of ∼133 mA h g^–1^ by Fe^2+^/Fe^3+^ (∼2.5 V), V^3+^/V^4+^ (∼3.4 V), and V^4+^/V^5+^ (∼4.0 V) redox couples, corresponding to 2.4 mol electron exchange per formula unit (Figure 2H).^24^ More recently, Masquelier et al. successfully prepared a Na_4_VFe(PO_4_)_3_ cathode with a Fe/V atom ratio of 1:1.^25^ The cathode allows an electrochemical extraction of 2.76 Na^+^ but only a reversible insertion of ∼2.4 Na^+^ per formula unit due to the irreversibility of the V^4+^/V^5+^ transition (Figure 2I).^25^ Regarding the origin of the irreversible V^4+^/V^5+^ redox, two possible mechanisms from the perspectives of the structural aspects are summarized here to provide some thinking direction for further investigation. The first could be associated with the extraction of Na^+^ from the Na1 sites. Upon charging to high voltage (usually above 3.8 V), the inert Na^+^ located at the Na1 sites was forced to dislodge (i.e., the third Na^+^ extracted from the structure), which would lead to changed local environments and distortion of V^5+^O_6_ octahedra. The structure mismatch during charge and discharge process contributed to the irreversible electrochemical behavior. The other lies in the possible migration of highly oxidized and small V^5+^ ions to Na vacancies or other thermodynamically stable positions, resulting in the kinetic deterioration and structural instability related to the V^4+^/V^5+^ reaction center. Strangely, as for Na_4_VFe(PO_4_)_3_, it could be observed that this cathode still retains the V^4+^/V^5+^ charge plateau in the next cycles, delivering the asymmetric electrochemical behavior between charge and discharge processes all along. Actually, the main problem of these compounds containing divalent Fe^2+^ lies in their susceptibility and instability in air during storage, which puts forward huge challenges for practical application. In view of this, more stable Mn^2+^ ions have drawn greater attention from researchers. Zakharkin et al. systematically investigated the electrochemical properties and phase transformation behavior in the NASICON-type Na_3+*x*_Mn_*x*_V_2-*x*_(PO_4_)_3_ (0 ≤ *x* ≤ 1) cathodes.^26^ Their findings demonstrated that the electrochemical behavior of the Na_3+*x*_Mn_*x*_V_2-*x*_(PO_4_)_3_ family is highly determined by the Mn^2+^ doping content and voltage window (Figure 2J).^26^ Within the voltage range of 2.5–3.8 V, an increase of Mn^2+^ doping content would contribute to voltage boost without significant capacity loss. This is because the Mn^2+^/Mn^3+^ reactive center could render a higher potential (∼3.6 V) than that of the V^3+^/V^4+^ redox couple (∼3.4 V). The extended voltage range of 2.5–4.1 V makes the further oxidation of V^4+^ → V^5+^ or Mn^3+^ → Mn^4+^ in Na_3+*x*_Mn_*x*_V_2-*x*_(PO_4_)_3_ compounds possible for access to the three-electron reaction. However, as Mn content is low (x ∼ 0–0.4), only V^4+^/V^5+^ redox couples could be reached. Such a reversible Na deinsertion is still limited in the two-electron reaction. In the cases of Na_3.75_Mn_0.75_V_1.25_(PO_4_)_3_^31^ and Na_3.5_Mn_0.5_V_1.5_(PO_4_)_3_^32^ cathodes with higher Mn contents, the *ex situ* X-ray absorption spectra (XAS) indicate the formation of mixed V^4+^/V^5^ and Mn^3+^/Mn^4+^ redox in the deep desodiated states. Interestingly, however, the recent work reported by Masquelier’ group suggested only V^4+^ → V^5+^ transformation but without Mn^3+^ → Mn^4+^ oxidation in the high-voltage region of Na_4_MnV(PO_4_)_3_ electrode.^29^ The large capacity loss due to irreversible V^4+^/V^5+^ redox couples leads to the failures of the three-electron reaction in Na_4_MnV(PO_4_)_3_. Their investigations show that the irreversible electrochemical behavior in the high-voltage region could be ascribed to the distortion of Mn and V structural environments. Note that the V^4+^/V^5+^ charge plateau in Na_4_MnV(PO_4_)_3_ vanished from the second cycle, which is obviously different from the above-discussed Na_4_FeV(PO_4_)_3_ cathode (Figure 2K).^25,29^ The structural origin of the abnormal electrochemical behavior remains elusive. Besides, from the second cycle, the Na_4_MnV(PO_4_)_3_ electrode revealed the tilted and solid-solution-like charge/discharge curves without any distinguished voltage plateaus, which could be ascribed to the irreversible structural degradation upon first charging to over 3.8 V. Therefore, Na_4_MnV(PO_4_)_3_ was usually cycled within the voltage window of 2.5–3.8 V to balance the capacity delivery and structural stability.^33^

To further enhance the electrochemical performance of Na_4_MnV(PO_4_)_3_ cathodes, extensive efforts for the substitution of Mn with other stabilizers were conducted in recent years. Zhao et al. utilized Fe^2+^ to replace half Mn^2+^ in the Na_4_MnV(PO_4_)_3_ to generate the novel Na_4_Mn_0.5_Fe_0.5_V(PO_4_)_3_, which enables enhanced electrochemical performance due to synergistic effects from the multimetal ions.^28^ This ternary phosphate integrated the advantages of the large reversible capacity of the V-based phosphate cathode, the high voltage of Mn^2+^/Mn^3+^ redox couples, and the good stability of compatible Fe substitution. Furthermore, their theoretical calculation results indicate that, owing to the synergistic effect from the V, Fe, and ions, Na_4_Mn_0.5_Fe_0.5_V(PO_4_)_3_ simultaneously exhibits favorable electron conductivity and high redox activity in contrast to both binary Na_4_MnV(PO_4_)_3_ and Na_4_FeV(PO_4_)_3_, finally contributing to favorable kinetic properties and thereby improved electrochemical performance (Figure 2L). Ghosh and co-workers selected Mg^2+^/Al^3+^ as doping ions to reduce Mn content in the Na_4_MnV(PO_4_)_3_ cathodes.^34^ Owing to the enhanced bond energy between transition metal and oxygen, as well as the suppressed Jahn–Teller effect, the final doped cathodes delivered improved cycling stability. However, these strategies shorten the length of the voltage plateau attributed by the Mn^2+^/Mn^3+^ redox couple, which is accompanied by a decrease of voltage output and energy density. Under the premise of enabling the full utilization of Mn^2+^/Mn^3+^ in Na_4_MnV(PO_4_)_3_, rather than Mn sites, Al^3+^ was selectively doped in V sites to reduce the cost, which allows favorable kinetics and enhanced structural stability due to an enlarged diffusion channel and suppressed Jahn–Teller effect.^35^

### 2.2 Vanadium-Based Mixed Phosphates

#### NaVPO_4_X (X = O, F)

**Figure 3:**
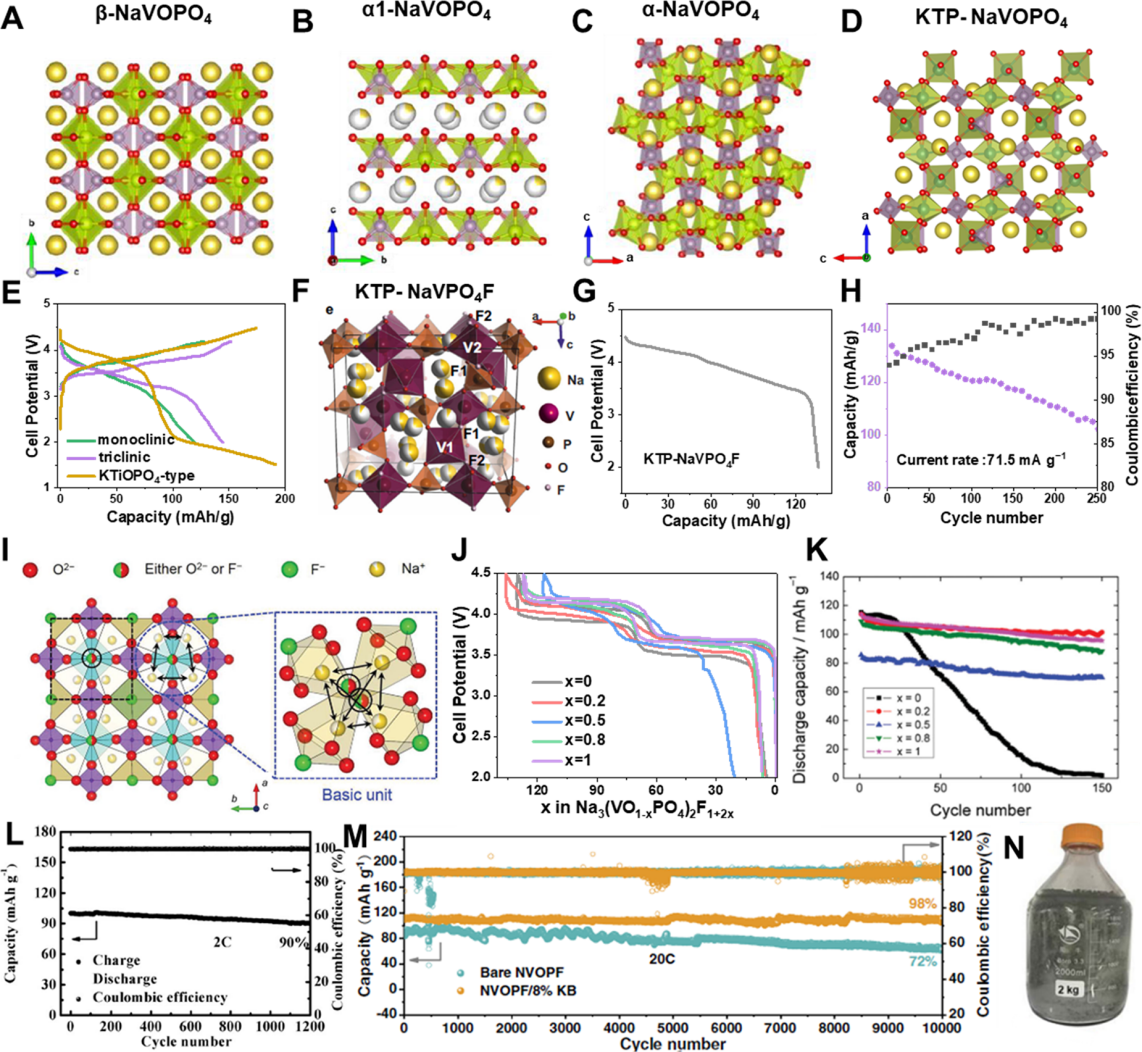
Schematic illustration of the crystal structures of four NaVOPO_4_ polymorphs: β-NaVOPO_4_ (A), α1-NaVOPO4 (B), α-NaVOPO_4_ (C), and KTiOPO_4_-type NaVOPO_4_ (D). Reproduced with permission from ref (37). Copyright 2016 American Chemical Society. (E) Charge/discharge profiles comparison of NaVOPO_4_ between different polymorphs, data from refs (39, 40, and 42). (F) Illustration of the crystal structures of KTiOPO_4_-type NaVPO_4_F. Typical charge/discharge profiles at 0.1 C (G) and cycling performance (H) of KTiOPO_4_-type NaVPO_4_F. Reproduced with permission from ref (43). Copyright 2022 Nature Publishing Group. (I) Crystal structure of Na_3_(VO_1–*x*_PO_4_)_2_F_1+2*x*_ (0 ≤ *x* ≤ 1). (J) Charge/discharge profiles comparison of Na_3_(VO_1–*x*_PO_4_)_2_F_1+2*x*_ (*x* = 0, 0.2, 0.5, 0.8, and 1). (K) Cycling performance of Na_3_(VO_1–*x*_PO_4_)_2_F_1+2*x*_ at a 0.5 C rate with a voltage window of 2.0–4.5 V. Reproduced with permission from ref (49). Copyright 2014 Wiley-VCH. (L) Cycling performance of hydrothermal synthetic Na_3_V_2_(PO_4_)_2_F_3_ at 2C. Reproduced with permission from ref (52). Copyright 2015 Wiley-VCH. (M) Cycling performance of the mechanochemically synthesized Na_3_(VOPO_4_)_2_F/KB nanoparticles at 20 C. (N) The as-synthesized kilogram-scale Na_3_(VOPO_4_)_2_F/8% KB products. Reproduced with permission from ref (57). Copyright 2021 Nature Publishing Group.

The combination of slight anions (e.g., O^2–^ and F^–^) with phosphate group was an effective strategy to increase the energy density of vanadium-based cathodes due to access to more reversible capacity.^7^ For instance, the NaVOPO_4_ member allows a theoretical capacity as high as 145 mA h g^–1^, showing a huge application potential. Up to now, NaVOPO_4_ cathodes were reported to crystalline in many different structures, exemplified by α-NaVOPO_4_ (monoclinic),^36^ β-NaVOPO_4_ (orthorhombic),^37^*α*_*1*_-NaVOPO_4_ (tetragonal),^38^ and triclinic NaVOPO_4_.^39^ Among them, orthorhombic (Figure 3A) and tetragonal NaVOPO_4_ (Figure 3B) cathodes could only be obtained by chemical sodiation of the delithiated VOPO_4_ intermediates, which makes the practical application of NIBs cost-ineffective and time-consuming because of the complex delithiation or presodiation process. In 2013, Goodenough’s group adopted a direct sol–gel method to successfully prepare the monoclinic NaVOPO_4_ cathodes (Figure 3C).^36^ But the material delivered a reversible capacity of only ∼90 mA h g^–1^ due to the kinetics limit. Recently, Zhao et al. synthesized the flower-like α-NaVOPO_4_ cathodes by a simple hydrothermal route.^40^ Thanks to the improved kinetics rendered by the nanoscale sizes and the multidimensional carbon network, NaVOPO_4_/C composites enable a large initial reversible capacity of 140.2 mA h g^–1^ (Figure 3E) and a decent capacity retention of 84.8% at 10 C after 1000 cycles. Such monoclinic phases usually have tunnel structures in which Na ions are zigzagged to transport through the lattice frameworks, leading to slow kinetics. For comparison, the NaVOPO_4_ cathode with a two-dimensional layered structure displays a better Na storage performance. Cao and co-workers applied a topochemical route to prepare the triclinic NaVOPO_4_ with layered structure using the VOPO_4_·2H_2_O as structural framework.^39^ The layered NaVOPO_4_ cathode could achieve an impressive reversible capacity of 144 mA h g^–1^ (theoretical value) with an average voltage of ∼3.5 V at 0.05 C within 2.0–4.3 V (Figure 3E), delivering an energy density of 465 W h kg^–1^ (hereinafter calculated by integrating the area of the charge/discharge curve). However, the high-rate capability and cycling performance need further improvement. Their group then reported an amorphous NaVOPO_4_ that served as a new cathode for NIBs.^41^ Although the reversible capacity decreased to 110 mA h g^–1^, a promising capacity retention of 96% could be retained when the reaction cycled at 10 C over 2000 cycles. Another form of NaVOPO_4_ is isostructural to the KTiOPO_4_-type family (Figure 3D),^42^ which was first reported by Whittingham’s group. The NaVOPO_4_ was synthesized by an ion-exchange route using the KTiOPO_4_-type NH_4_VOPO_4_ as mother structure. The obtained NaVOPO_4_ displayed a large discharge capacity of ∼200 mA h g^–1^ through utilization of a V^5+^/V^4+^/V^3+^ multielectron reaction (Figure 3E), but the V^4+^/V^3+^ redox couples in the low voltage range contributed about half of the capacity.

Utilization of F^–^ with strong electronegativity to replace O^2–^ in NaVOPO_4_ enables a higher voltage output of the materials due to the tunable inductive effect. As a typical case, the KTiOPO_4_-type NaVPO_4_F cathodes (Figure 3F) delivered a 4 V-level average voltage and reversible capacity of 136 mA h g^–1^, showing a promising energy density of ∼540 W h kg^–1^ (Figure 3G).^43^ However, the extended voltage window between 2.0 and 4.5 V has a negative impact on the stability of an organic electrolyte and the tolerance of materials’ structure, leading to a relatively inferior cycle lifespan (Figure 3H). Further nanoengineering or carbon coating strategies are urgent and imperative to improve their cycling stability. Apart from the new KTiOPO_4_-type structure, NaVPO_4_F was also crystallized to other polymorphs, i.e., monoclinic^44^ and tetragonal^45^ phases. The tetragonal NaVPO_4_F with a space group of *I*4*/mmm* was first proposed by Barker’ group, which exhibited an average discharge voltage of 3.7 V.^45^ Subsequently, Zhuo et al. synthesized a monoclinic NaVPO_4_F (*C*2/*c* space group), but this positive electrode only displayed 3.4 V average voltage.^44^ Zheng et al. found that tetragonal NaVPO_4_F would transform into the monoclinic after a post-calcination above 600 °C.^46^ This means that the tetragonal NaVPO_4_F with a higher voltage output could have a poorer thermal stability than that in the monoclinic phase. However, the tetragonal NaVPO_4_F showed similar XRD patterns with Na_3_V_2_PO_4_F_3_ (to be discussed in the following part); meanwhile, the structure of monoclinic NaVPO_4_F is nearly the same as that of Na_3_V_2_(PO_4_)_3_. These striking similarities make researchers cast doubt on the accuracy of the previously obtained sodium vanadium fluorophosphates with nominal composition of NaVPO_4_F. Li et al. believed that the so-called “NaVPO_4_F” from solid-state synthesis could be the mixtures of Na_3_V_2_(PO_4_)_2_F_3_, VPO_4_, and Na_3_V_2_(PO_4_)_3_, which is concluded from the analysis of the chemical preparation process and structural evolution by *in situ* XRD and thermal characterizations.^47^ Up to now, there has still been significant controversy over the crystal structure of such “NaVPO_4_F” materials.

#### Na_3_(VO_1–*x*_PO_4_)_2_F_1+2*x*_ (0 ≤ *x* ≤ 1)

Some other well-known sodium vanadium fluorophosphates belong to the Na_3_(VO_1–*x*_PO_4_)_2_F_1+2*x*_ (0 ≤ *x* ≤ 1) family, which usually demonstrates a high energy density of ∼500 W h kg^–1^ with good cycling stability.^7,48^ By tuning the atomic ratio (x value), the local structure and electrochemical properties of the Na_3_(VO_1–*x*_PO_4_)_2_F_1+2x_ compounds could be of targeted regulation (Figure 3I). With the increase of F^–^, the materials delivered an elevated voltage plateau. On the contrary, more incorporated O^2–^ in the compounds enables increased Na^+^ disorder and tilted voltage-capacity profile. Despite these differences, Kang et al. found that a series of Na_3_(VO_1–*x*_PO_4_)_2_F_1+2*x*_ (*x* = 0, 0.2, 0.5, 0.8, 10) compounds showed analogous charge/discharge curves, all of which revealed the average voltages of over 3.8 V and large reversible capacities and reasonable capacity retention (Figure 3J-K).^49^ In the Na_3_(VO_1–*x*_PO_4_)_2_F_1+2*x*_ (0 ≤ *x* ≤ 1) family, two end members of Na_3_V_2_(PO_4_)_2_F_3_ and Na_3_(VOPO_4_)_2_F were most researched. Na_3_V_2_(PO_4_)_2_F_3_ was first reported to be crystalline in the tetragonal phase with a space group of *P*4_2_*/mnm*, in which V_2_O_8_F_3_ polyhedrons were alternately bridged by PO_4_ tetrahedrons.^49^ But Bianchini et al. believed that the structure of Na_3_V_2_(PO_4_)_2_F_3_ belongs to an orthorhombic phase with the *Amam* space group, which demonstrated different Na-site distributions from the tetragonal one.^50^ Due to the different synthesis routes and characterization methods, there are some distinctions in terms of the electrochemical reaction mechanism during de/sodiation. Before 2015, it was believed that Na_3_V_2_(PO_4_)_2_F_3_ transformed into NaV_2_(PO_4_)_2_F_3_ through one step solid-solution reaction. However, Bianchini et al. found that the Na_3_V_2_(PO_4_)_2_F_3_ cathode could experience a multiphase transition accompanied by generation of two intermediate states of Na_2.4_V_2_(PO_4_)_2_F_3_ and Na_2.2_V_2_(PO_4_)_2_F_3_.^51^ For Na_3_(VOPO_4_)_2_F, it was generally believed to belong to the tetragonal phase (*I*4*/mmm*).^52^ Similar to that of Na_3_V_2_(PO_4_)_2_F_3_, both solid-solution reaction and biphase transformation have been demonstrated in previous reports.^53^ Regardless of the different reaction mechanism, Na_3_(VOPO_4_)_2_F serving as cathodes for NIBs showed a highly reversible electrochemical reaction evolution, revealing a small volume variation of only 2.56% during the charge/discharge process.^53^

The mystery of the synthesis of Na_3_(VO_1–*x*_PO_4_)_2_F_1+2*x*_ (0 ≤ *x* ≤ 1) compounds can be summarized as a development history from high temperature to low temperature and from complexity to simplicity. Before 2014, Na_3_(VO_1–*x*_PO_4_)_2_F_1+2*x*_ members were usually prepared by a high-temperature solid-state method using stoichiometric contents of VOPO_4_/VPO_4_, NaF, and Na_2_CO_3_ as reaction feeds.^49^ However, such an energy-consuming high-temperature calcination not only increases the cost of materials production but also easily leads to equipment damage due to the strong corrosion of fluorides. Considering this, Zhao et al. adopted a solvothermal low-temperature strategy to successfully prepare a series of Na_3_(VO_1–*x*_PO_4_)_2_F_1+2*x*_ (*x* = 0, 0.3, 0.5, 0.7, 0.9, 1) compounds with a high-purity phase.^52^ The nanosized samples of Na_3_V_2_(PO_4_)_2_F_3_ without any carbon coating enable a decent capacity retention of over 90% after 1200 cycles at 2 C (Figure 3L). Subsequently, Guo’s group prepared the Na_3_(VOPO_4_)_2_F nanocubes by a hydrothermal method using V_2_O_5_, H_2_C_2_O_4_, NH_4_H_2_PO_4_, and NaF as the starting materials.^53^ The target Na_3_(VOPO_4_)_2_F cathode demonstrated a superior low-temperature performance and high energy density in the full cells. Zhao et al. systematically investigated the correlations between various reaction conditions, micromorphology, and the Na-storage performance in the solvothermal reaction system.^54^ The results indicate the raw materials, pH value, reaction time, and solvent types played key roles on the microshape, particle size, phase purity, and thereby electrochemical performance of the final cathodes. Such a comprehensive study offers directional guidance for the design of the Na_3_(VO_1–*x*_PO_4_)_2_F_1+2*x*_ family with controllable microarchitectures and desirable electrochemical performance. To reduce the energy cost, our group further developed a facile one-step room-temperature strategy for the scalable fabrication of Na_3_(VOPO_4_)_2_F cathodes.^55^ Benefiting from the soft templates of the hydroxylamine reducing agent and the controlled release of vanadium, the kg-level Na_3_(VOPO_4_)_2_F multishelled microspheres could be spontaneously generated in a room-temperature condition, which allows a good capacity retention of 70% after 3000 cycles at a high current rate of 15 C. After that, through the use of the NH_3_·H_2_O as reaction accelerator, the monodispersed Na_3_(VOPO_4_)_2_F submicrometer cubes were rapidly and massively produced, delivering an excellent cycling performance of 8000 times at 20 C.^56^ More recently, to promote the production efficiency and electrochemical performance, Zhao et al. proposed a rapid and solvent-free mechanochemical synthesis strategy (only 30 min) to prepare the carbon-coated Na_3_(VOPO_4_)_2_F nanocomposites.^57^ The target products demonstrated superior electrochemical performance due to construction of effective carbon network and nanoscale particle size, delivering an impressive capacity retention of ∼98% over 10000 times at 20 C (Figure 3M). Moreover, 2 kg of Na_3_(VOPO_4_)_2_F products (Figure 3N) were employed to fabricate the 26650-prototype batteries, enabling good capability, low-temperature performance, and cycling stability, which marks an important step in the industrial application of sodium vanadium fluorophosphates for NIBs.

## 3 Iron Based Polyanionic Compounds

### 3.1 Iron-Based Phosphates

#### Phosphates

**Figure 4:**
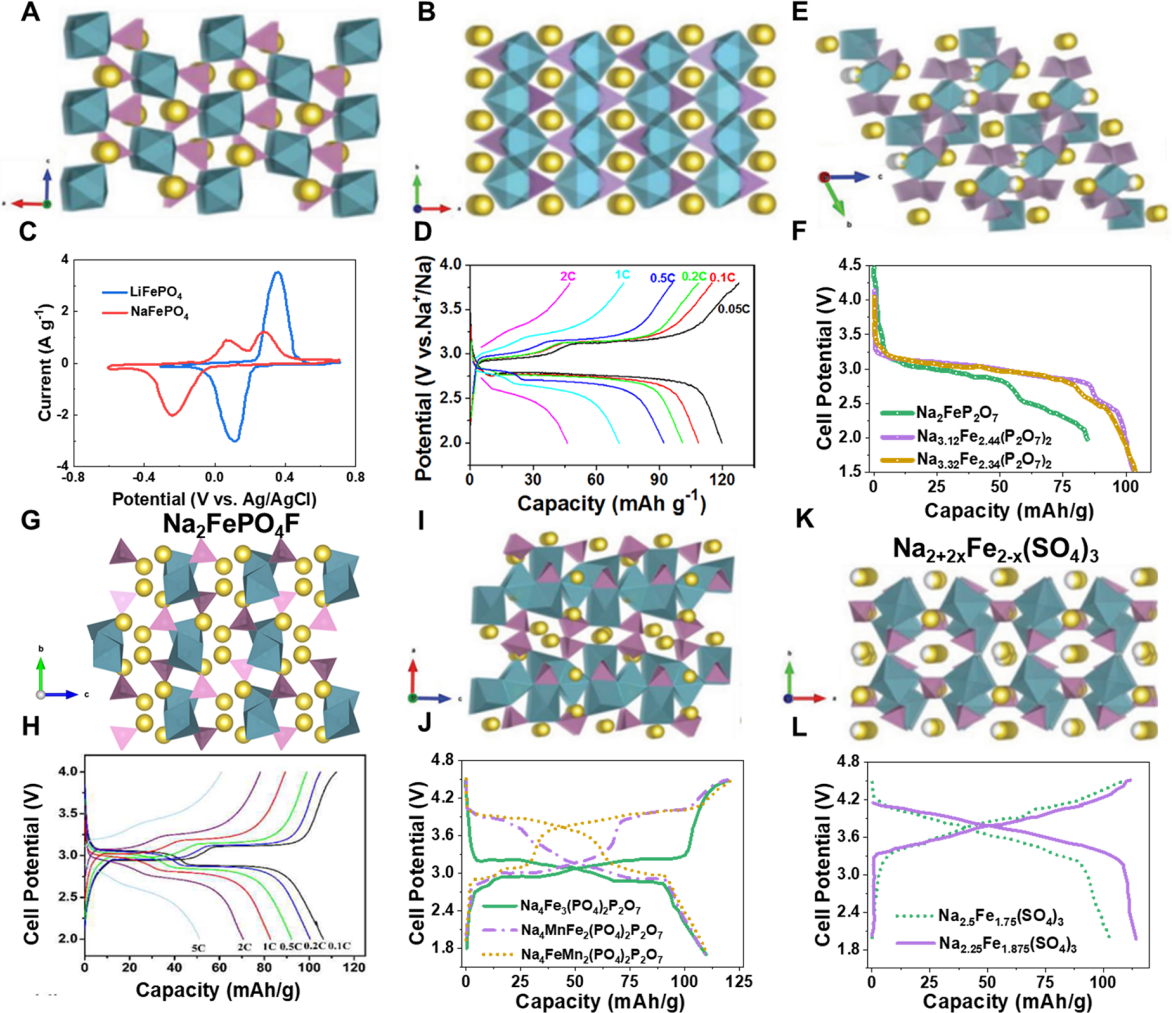
Schematic representations of maricite (A) and triphylite (B) NaFePO_4_, (E) Na_2_FeP_2_O_7_, (G) Na_2_FePO_4_F, (I) Na_4_Fe_3_(PO_4_)_2_P_2_O_7_ and (K) Na_2+2*x*_Fe_2–*x*_(SO_4_)_3_. Reproduced with permission from ref (5). Copyright 2019 Wiley-VCH. (C) Cyclic voltammetry of the aqueous electrochemical displacement process from triphylite LiFePO_4_ to FePO_4_ in 1 M Li_2_SO_4_ solution and then to NaFePO_4_ in 1 M Na_2_SO_4_ solution. (D) Voltage-capacity profiles of the NaFePO_4_/C electrode at different charge/discharge rates from 0.05 to 2 C. Reproduced with permission from ref (61). Copyright 2015 American Chemical Society. (F) Discharge profiles comparison between Na_2_FeP_2_O_7_, Na_3.12_Fe_2.44_(P_2_O_7_)_2_ and Na_3.32_Fe_2.34_(P_2_O_7_)_2_, data from ref (63, 65, and 66). (H) Voltage-capacity profiles of the Na_2_FePO_4_F/C at different charge/discharge rates. Reproduced with permission from ref (72). Copyright 2018, Elsevier. (J) Charge/discharge profiles comparison between Na_4_Mn_*x*_Fe_3–*x*_(PO_4_)_2_(P_2_O_7_) (*x* = 0, 1, 2), data from ref (74). (L) Charge/discharge profiles of Na_2.5_Fe_1.75_(SO_4_)_3_ and Na_2.25_Fe_1.875_(SO_4_)_3_, data from refs (81 and 83).

The success of LiFePO_4_ for LIBs sparked extensive research interest to prepare NaFePO_4_ cathodes for NIBs. Unfortunately, the direct synthesis of the triphylite NaFePO_4_ has not been reported so far, since the triphylite structure of NaFePO_4_ is considered to be a thermodynamic unstable phase.^7,58^ On the contrary, the maricite NaFePO_4_ was proven to be thermodynamically favored, but no Na^+^ diffusion channels could be observed in its structure (Figure 4A). The electrochemical activity of the maricite-type NaFePO_4_ can be activated through downsizing/nanoengineering and the construction of amorphous structures; however, most of the capacity is contributed from the low voltage region and is therefore not attractive.^59,60^ The triphylite-type NaFePO_4_ is usually prepared by electrochemical sodiation of the delithiated LiFePO_4_ (Figure 4B), which undergoes a phase-transition during the charging process and obvious voltage drop during the discharging process (Figure 4C), finally leading to undesirable electrochemical performance and low energy density (Figure 4D).^61^ Besides, the complex synthesis route also limits its practical application. The NASICON-type Na_3_Fe_2_(PO_4_)_3_ is not suitable as a cathode for practical NIBs because the Na-free anode (usually hard carbon) cannot render extra Na^+^ to activate the Fe^2+^/Fe^3+^ redox.^62^ Moreover, the voltage plateau for Fe^2+^/Fe^3+^ redox in the NASICON phosphates is low (∼2.5 V), greatly limiting the energy density. Further activation of the Fe^3+^/Fe^4+^ redox couple is expected in Na_3_Fe_2_(PO_4_)_3_, but more likely, its potential is too high to be achieved within the proper voltage window considering the possible electrolyte oxidation.

#### Pyrophosphates

Na_2_FeP_2_O_7_ was considered to be a safer cathode than the phosphates owing to its higher thermal stability. Na_2_FeP_2_O_7_ has a triclinic structure and belongs to the *P1* space group, where FeO_6_ octahedra and P_2_O_7_ groups are connected by corner or edge-sharing, thus forming a three-dimensional framework with spacious Na^+^ channels (Figure 4E).^63^ The Na storage performance of the Na_2_FeP_2_O_7_ cathode was revealed by Yamada’s group. The cathode delivered a reversible capacity of ∼90 mA h g^–1^ through a one-electron reaction based on the Fe^2+^/Fe^3+^ redox couples (Figure 4F).^63^ The stable and open framework renders the decent cycling performance and rate capability of the Na_2_FeP_2_O_7_ cathode for NIBs; nevertheless, practical application could be hindered by its low energy density. One can easily discover that utilization of only half of sodium in Na_2_FeP_2_O_7_ is one direct reason for its low energy density. In response, Nazar’s group proposed a new solid solution series Na_4-α_Fe_2+α/2_(P_2_O_7_)_2_ (2/3 ≤ α ≤ 7/8) to increase the number of the electrochemically available Fe (or Na).^64^ Two representative cathodes of Na_3.12_Fe_2.44_(P_2_O_7_)_2_ and Na_3.32_Fe_2.34_(P_2_O_7_)_2_, as members of the Na_4-α_Fe_2+α/2_(P_2_O_7_)_2_ (α = 0.88 and 0.68, respectively) family, have higher theoretical capacities (∼117 mA h g^–1^) than Na_2_FeP_2_O_7_ (97 mA h g^–1^). Liu et al. successfully prepared the Na_3.12_Fe_2.44_(P_2_O_7_)_2_ cathode via conventional solid-state technique.^65^ The obtained products enabled a reversible capacity of ∼105 mA h g^–1^ (Figure 4F). Chen et al. prepared a carbon-coated Na-rich Na_3.32_Fe_2.34_(P_2_O_7_)_2_, which delivered an initial capacity of 107 mA h g^–1^ (Figure 4F).^66^ These findings further confirm that construction of solid-solution Na_4-α_Fe_2+α/2_(P_2_O_7_)_2_ by the off-stoichiometric strategy indeed increases the energy density (specific capacity), making the pyrophosphate cathodes more of a prospect.

#### Mixed Phosphates

The phosphate group combined with the electronegative F^–^ could increase voltage output due to a changed inductive effect. Na_2_FePO_4_F crystallizes in an orthorhombic structure with the space group of *Pbcn*, in which the face-sharing FeO_4_F_2_ octahedra were bridging connected by F atoms and PO_4_ tetrahedra to form the [FePO_4_F] layers, thus providing two-dimensional Na^+^ ion transport paths (Figure 4G).^67^ Based on the Fe^2+^/Fe^3+^ redox couples, a theoretical capacity of 124 mA h g^–1^ and an average voltage of 3.0 V can be obtained by removal of 1 mol of Na^+^ from Na_2_FePO_4_F for NIBs. Due to the generation of the intermediate Na_1.5_FePO_4_F phase, two distinct voltage plateaus appear in the charge/discharge curves.^68^ However, the electrochemical performance of Na_2_FePO_4_F is not satisfactory from the summary of the literature,^69,70^ which could be ascribed to the poor intrinsic conductivity and unstable layered structure. To boost the voltage output of Na_2_FePO_4_F-based cathodes, Mn^2+^ doping was attempted to obtain the solid-solution Na_2_Fe_1–*x*_Mn_*x*_PO_4_F compounds.^71^ Unfortunately, the Mn doping seems to be adverse for the increase in energy density due to the significant capacity loss, because incorporation of Mn into the materials would induce a structure transformation from the layered to tunnel phase, leading to the deactivation of Fe/Mn elements. Recently, Cai et al. proposed a porous carbon coating strategy to enhance the electrochemical performance of the Na_2_FePO_4_F (Figure 4H).^72^ Thanks to the reduced particle size and appropriate carbon coating, the optimal samples demonstrate enhanced kinetics and rate capability, but the cycling stability needs further improvement.

Compared with individual Na_2_FeP_2_O_7_ or NaFePO_4_, the mixed phosphates Na_4_Fe_3_(PO_4_)_2_P_2_O_7_ exhibited more application potentials. Na_4_Fe_3_(PO_4_)_2_P_2_O_7_ crystallizes in the orthorhombic structure with the space group of *Pn*2_1_*a*, which is composed of [Fe_3_P_2_O_13_]∞ infinite layers parallel to the b-c plane (Figure 4I).^73^ Its Na storage performance was revealed by Kang’s group for the first time in 2012.^73^ Through the Fe^2+^/Fe^3+^ reaction, Na_4_Fe_3_(PO_4_)_2_P_2_O_7_ allows a theoretical capacity of over 110 mA h g^–1^ with an average voltage of 3.1 V, far exceeding the iron-based phosphates or pyrophosphates (Figure 4J). By replacing Fe with Mn, the binary mixed-phosphate phase of Na_4_Mn_*x*_Fe_3*–*x_(PO_4_)_2_(P_2_O_7_) (*x* = 1 or 2) demonstrating higher voltage output due to the high potential of the Mn^2+^/Mn^3+^ redox couples (Figure 4J).^74^ Despite the elevated energy density, Na_4_Mn_*x*_Fe_3–*x*_(PO_4_)_2_(P_2_O_7_) delivered a compromised cycling performance due to a deteriorated dynamic performance related to Mn. Another form of the mixed iron-based phosphates-pyrophosphates is Na_3_Fe_2_PO_4_P_2_O_7_, rendering a theoretical capacity of ∼120 mA h g^–1^ based on Fe^2+^/Fe^3+^ redox couples, which was first reported by Xia’s group,^75^ However, the synthesis conditions need to be carefully controlled (e.g., calcination temperature and gas atmosphere), because it is easy to obtain the more stable NASICON-type Na_3_Fe_2_(PO_4_)_3_ phase as discussed due to the same reactants.

### 3.2 Iron-Based Sulfates

#### Bisulfates

Inspired by the high redox potential of Fe^2+^/Fe^3+^ (3.6–3.9 V vs. Li/Li^+^) in Li-based sulfates (e.g., Li_2_Fe(SO_4_)_2_) stemming from the electronegativity of SO_4_^2–^,^76^ parallel efforts were devoted to exploring the Na-based bisulfate candidates. However, due to the high sensitivity to moisture, the Na-based bisulfates tend to stably exist in the form of their hydrated phases. Na_2_Fe(SO_4_)_2_·4H_2_O crystallizes in a monoclinic phase with the space group of *P2*_1_/*c*, which could be prepared by precipitation of Na_2_SO_4_ and FeSO_4_·7H_2_O in aqueous media using alcohol.^77^ Alternatively, without using alcohol in the reaction system, Na_2_Fe(SO_4_)_2_·2H_2_O was preferentially generated.^78^ Both Na_2_Fe(SO_4_)_2_·4H_2_O and Na_2_Fe(SO_4_)_2_·2H_2_O were found to be electrochemically active and served as cathodes for NIBs, which could deliver reversible capacities of ∼50 and 70 mA h g^–1^, respectively. The average potentials of Fe^3+^/Fe^2+^ redox couples in these compounds were about 3.3 V. Different from the amorphization of crystal structure in Na_2_Fe(SO_4_)_2_·4H_2_O, Na_2_Fe(SO_4_)_2_·2H_2_O demonstrates reversible structure evolution and high stability during the charging/discharging process. The anhydrous Na_2_Fe(SO_4_)_2_ could be easily obtained by removal of the crystal water from Na_2_Fe(SO_4_)_2_·4H_2_O or Na_2_Fe(SO_4_)_2_·2H_2_O via vacuum heating treatment.^77^ However, the electrochemical performance of Na_2_Fe(SO_4_)_2_ is not desirable, revealing an average output of 3.4 V but a low capacity delivery of ∼70 mA h g^–1^. Goodenough’s group reported a Fe^3+^-based NaFe(SO_4_)_2_, which belongs to a monoclinic structure with *C*2/*m* symmetry and is structurally different from the above-mentioned bisulfates.^79^ Due to the less molar weight caused by the Na defects, its discharge capacity slightly increased to 80 mA h g^–1^ based on the single-phase redox mechanism. However, the limited capacity and Na-deficiency features hinder its practical use.

#### Trisulfates

There have been reported two types of trisulfates, i.e., NASICON and alluaudites.^80^ The NASICON-type structure was believed to be a desirable ion conductor; however, the Fe_2_(SO_4_)_3_ in the rhombohedral NASICON phase exhibited sluggish kinetics because the small size of SO_4_^2–^ anions limits the site availability and the mobility of Na^+^ ions. As a contrast, Na_2_Fe_2_(SO_4_)_3_ with alluaudite polymorph delivered highly electrochemical activity due to facilitated ion diffusion pathway, which was first reported by Yamada’s group.^81^ The structure of Na_2_Fe_2_(SO_4_)_3_ alluaudite is generally considered a monoclinic framework with a space group of *C*2/*c* symmetry, in which the Fe_2_O_10_ dimers were abridged by SO_4_ tetrahedra to construct an open framework (Figure 4K). Serving as a cathode for NIBs, Na_2_Fe_2_(SO_4_)_3_ alluaudite enables a reversible capacity over 100 mA h g^–1^ and 3.8 V-level voltage output (Figure 4L), showing huge application potential. Theoretically, Na_2_Fe_2_(SO_4_)_3_ has a capacity of 120 mA h g^–1^; however, a sodium-rich Na_2+2*x*_Fe_2–*x*_(SO_4_)_3_ (*x* ∼ 0.25) was obtained instead of the stoichiometric compound Na_2_Fe_2_(SO_4_)_3_ by a ceramic method, which makes its real capacity decrease to 100 mA h g^–1^.^82^ To reduce the value of *x* to increase the capacity, Zhao et al. prepared a target Na_6_Fe_5_(SO_4_)_8_, one member of Na_2+2*x*_Fe_2–*x*_(SO_4_)_3_ in the case of *x* = 0.125, which showed a reversible capacity of ∼110.2 mA h g^–1^ and superior cycling stability (Figure 4L).^83^ Although some solution-based routes have been attempted to further lower *x* to 0, the electrochemical performance of the final cathodes was not desirable, which could be ascribed to the intrinsic structural defects induced by the hygroscopicity in an aqueous environment.^84^ Another issue lies in the presence of the SO_4_^2–^ group, which makes alluaudites prone to moisture attack and chemical degradation in the air, which greatly hinders its long-term storage and fabrication of electrodes. To address the humidity sensitivity of Na_2_Fe_2_(SO_4_)_3_ alluaudite, various carbon coating strategies were introduced as protective barriers against water attack. Rojo et al. utilized Ketjen Black carbon and reduced graphene oxide as both conductive carbon and water inhibitor of Na_2+2*x*_Fe_2–*x*_(SO_4_)_3_ compounds.^85^ As a result, the carbon-protected cathodes exhibited no water absorption peaks after exposure in the moist air for 24 h. Despite these advancements, realization of air stable and stoichiometric Na_2_Fe_2_(SO_4_)_3_ cathodes in the future still needs more effort and breakthrough from intrinsic structure.

#### Mixed Sulfates

Generally, NaFeSO_4_F belongs to the monoclinic phase with the space group of *C*2/*c*, which could be prepared by a topotactic reaction between NaF and FeSO_4_·H_2_O monohydrate precursors.^86^ However, NaFeSO_4_F serving as a cathode for NIBs showed poor electrochemical activity due to unfavorable kinetics and structural deformation for large Na^+^ diffusion. Kim et al. prepared a cation disordered NaFeSO_4_F by chemical sodiation of FeSO_4_F obtained by removal the Li^+^ from triplite LiFeSO_4_F.^87^ Although the resulting material delivered reasonable electrochemical activity and a high redox potential of ∼3.7 V, the complicated synthesis route makes it impossible to use in practical application. Tarascon’s group used the KTP-type KFeSO_4_F as the Na^+^ insertion host, which enables efficient Na^+^ (de)insertion leading to a capacity over 120 mA h g^–1^ with a flat stepwise voltage profile centered at 3.5 V.^88^ Despite the appealing electrochemical behavior, the pristine KTP-type NaFeSO_4_F cannot be directly synthesized and has not been reported yet. Other mixed sulfates of NaFe_2_PO_4_(SO_4_)_2_ proposed by Goodenough’s group were considered as potential cathodes for NIBs due to the low cost of raw materials and availability of Fe^2+^/Fe^3+^ redox couples.^89^ NaFe_2_PO_4_(SO_4_)_2_ crystallizes in a hexagonal NASICON structure and allows a two-electron reaction based on the Fe^2+^/Fe^3+^ reaction center with an average voltage of ∼3.0 V. As the trivalent Fe^3+^ existed in the as-prepared state, NaFe_2_PO_4_(SO_4_)_2_ was initially discharged to get the Na-rich Na_3_Fe_2_PO_4_(SO_4_)_2_ when cycled in the half cells. To get a practical application, Kumar et al. successfully prepared the Fe^2+^-based NASICON-typed Na_3_Fe_2_PO_4_(SO_4_)_2_ using (NH_4_)_2_Fe(SO_4_)_2_ as starting materials.^90^ However, the low energy density and poor cycling stability limit its further development.

## 4 Manganese Based Polyanionic Compounds

### 4.1 NASICON Manganese-Based Phosphates

#### Mn–Ti Compounds

**Figure 5:**
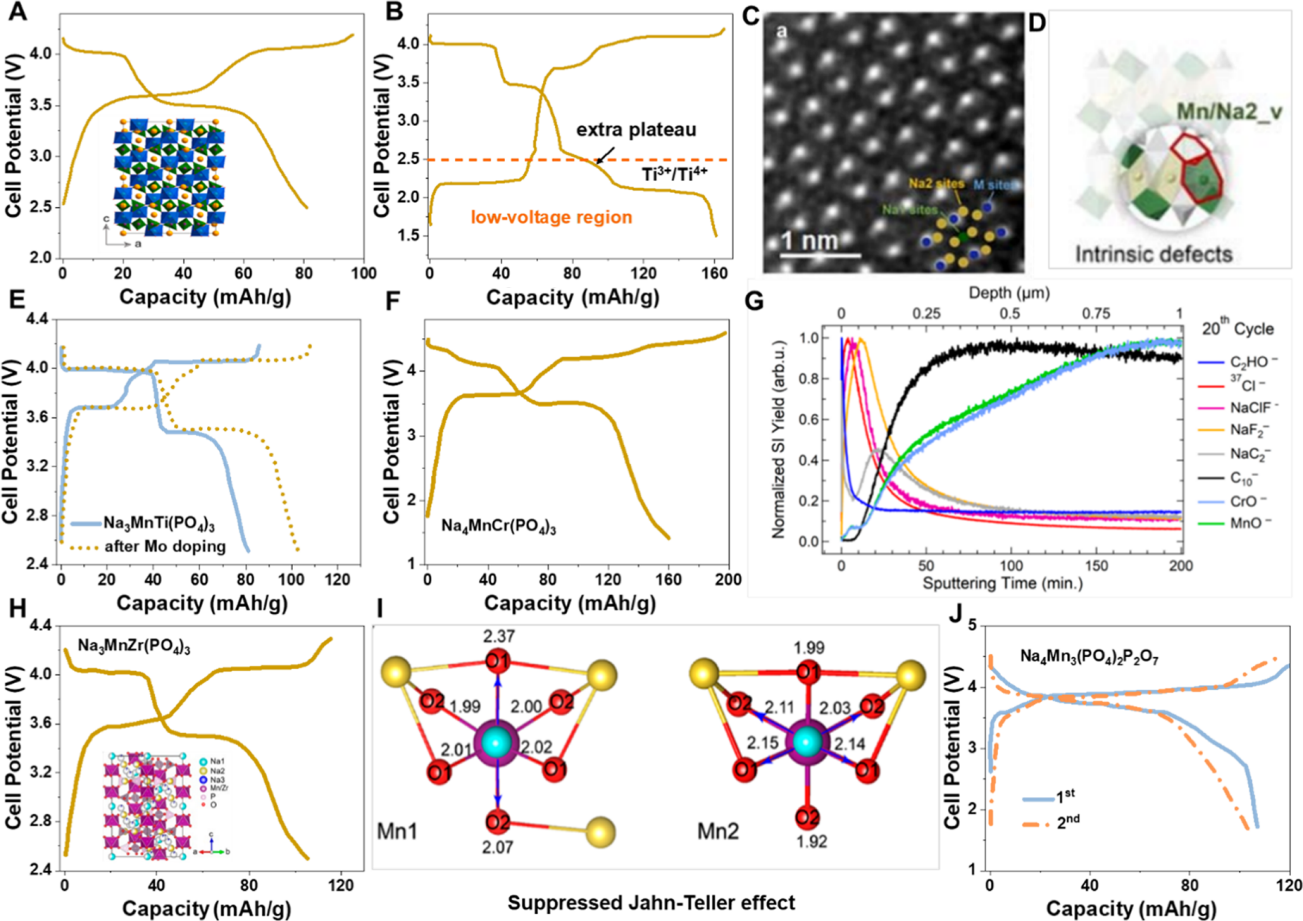
(A) Galvanostatic charge/discharge curves of the Na_3_MnTi(PO_4_)_3_ electrode at a rate of 0.1 C between 2.5 and 4.2 V. Inset is the structure illustration of Na_3_MnTi(PO_4_)_3_. Reproduced with permission from ref (91). Copyright 2016 American Chemical Society. (B) The charge/discharge curves of the Na_3_MnTi(PO_4_)_3_ between 1.5 and 4.2 V, data from ref (92). (C) Spherical-aberration STEM images of as-prepared Na_3_MnTi(PO_4_)_3_. (D) The illustration of intrinsic structure defects in Na_3_MnTi(PO_4_)_3_. (E) The charge/discharge curves comparison of Na_3_MnTi(PO_4_)_3_ before and after Mo doping. Reproduced with permission from ref (94). Copyright 2023 Nature Publishing Group. (F) The charge/discharge curves of the Na_4_MnCr(PO_4_)_3_ between 1.5 and 4.6 V, data from ref (98). (G) TOF-SIMS depth profiles of various species of interest obtained from the Na_4_MnCr(PO_4_)_3_ electrode after 50 cycles within the potential of 2.5–4.6 V. Reproduced with permission from ref (99). Copyright 2021 American Chemical Society. (H) The charge/discharge curves of the Na_3_MnZr(PO_4_)_3_ between 2.5 and 4.2 V. Inset is the structure illustration of Na_3_MnZr(PO_4_)_3_. (I) Coordination of two Mn sites, Mn1 and Mn2, in the lowest energy structure of Na_2_MnZr(PO_4_)_3_. Reproduced with permission from ref (101). Copyright 2018 American Chemical Society. (J) The initial two charge/discharge profiles of Na_4_Mn_3_(PO_4_)_2_P_2_O_7_ electrode, data from ref (104).

Na_3_MnTi(PO_4_)_3_ with a rhombohedral NASICON structure was first proposed by Hu’s group^1^ and synthesized as a cathode for NIBs by Goodenough’s group.^91^ Different from the above-discussed Na_4_MnV(PO_4_)_3_, theoretically, the multielectron reaction of Mn^2+^/Mn^3+^/Mn^4+^ could be achieved in Na_3_MnTi(PO_4_)_3_ due to the absence of irreversible structural deformation caused by the V^4+^/V^5+^ transition. However, the reversible capacity was delivered to be only 80 mA h g^–1^ from Na_3_MnTi(PO_4_)_3_ in the voltage range of 2.5–4.2 V (Figure 5A), which is obviously lower than the theoretical value (117 mA h g^–1^) rendered by the two-electron reaction through Mn^2+^/Mn^3+^ (3.6 V) and Mn^3+^/Mn^4+^ (4.1 V) redox couples. By extending the voltage window (1.5–4.2 V), Na_3_MnTi(PO_4_)_3_ enables a high specific capacity of 160 mA h g^–1^ at 0.2 C in a half cell (Figure 5B), which is attributed to the access to the third electron exchange by the extra utilization of the low-voltage Ti^3+^/Ti^4+^ (∼2.1 V) redox couples.^92^ It could be easily found that the low voltage region below 2.5 V contributes considerable discharge capacities, which include the Ti^3+^/Ti^4+^ reaction center and an abnormal plateau within 2.1–2.5 V. Of note, in a practical full cell, the capacity from the Ti^3+^/Ti^4+^ reaction center is usually unavailable, the main reasons being its low voltage and no extra Na insertion from the Na-free anode (hard carbon). In terms of the capacity contribution from 2.1–2.5 V, Zhang et al. considered that it was related to Na^+^/Mn^2+^ cationic mixing of Na_3_MnTi(PO_4_)_3_ cathodes, which not only lowers the effective capacity in the voltage range of 2.5–4.2 V, but also causes a severe charge/discharge voltage hysteresis, finally resulting in low energy efficiency.^93^ More recently, Hu’s group demonstrated that the intrinsic structure defects existed in the as-prepared Na_3_MnTi(PO_4_)_3_ (Figure 5C), in which the Mn^2+^ ions are preferably occupied on the Na vacancies (18 e) (Figure 5D), thus leading to the deteriorated kinetics properties and undesirable voltage hysteresis.^94^ Furthermore, they found that the samples doped by trace molybdenum could deliver the suppressed intrinsic structure defects and voltage hysteresis (Figure 5E). As a result, the modified cathodes exhibit two distinct Mn^2+^/Mn^3+^ and Mn^3+^/Mn^4+^ voltage plateaus, enabling the reversible capacity of ∼110 mA h g^–1^ between 2.5 and 4.2 V. Other strategies, such as vanadium doping,^95^ alkali excess^96^ routes, are also used to improve the electrochemical performance of Na_3_MnTi(PO_4_)_3_ to a certain extent. Considering the low cost and improved electrochemical performance by these effective strategies, further scale-up production and performance evaluation/optimization of Na_3_MnTi(PO_4_)_3_ pouch/cylindrical cells should be conducted as soon as possible to promote the application process of NIBs.

#### Mn–Cr Compounds

Na_4_MnCr(PO_4_)_3_ was considered one of the most promising phosphate cathodes due to its remarking energy density rendered by the three-electron reaction through Mn^2+^/Mn^3+^, Mn^3+^/Mn^4+^, and Cr^3+^/Cr^4+^ (4.5 V) redox couples.^97,98^ Na_4_MnCr(PO_4_)_3_ cathodes with the rhombohedral crystal structure were successfully prepared by Chen et al. via a simple sol–gel method, which enables a high reversible discharge capacity of 160.5 mA h g^–1^ and a decent average working voltage of 3.53 V between 1.5 and 4.6 V, corresponding to an ultrahigh energy density of 566.5 W h kg^–1^ (Figure 5F).^98^ However, one could observe that the three-electron reaction of Na_4_MnCr(PO_4_)_3_ is not completely reversible, especially the Cr^3+^/Cr^4+^ reaction center in the high voltage region. Such poor reversibility of Cr^3+^/Cr^4+^redox couples could be also found in other Cr-based Na_3_Cr_2_(PO_4_)_3_ cathodes, which could be due to structural deformation or possible electrolyte decomposition operated in high voltage above 4.5 V. By the comprehensive analysis of the interfacial properties evolution of the Na_4_MnCr(PO_4_)_3_ electrode, Zhao et al. considered that the liquid electrolyte decomposition at high potentials should be one of reasons for limited cycling stability and rate performance (Figure 5G).^99^ To explore the possible structural origin behind capacity fading at high voltage, Ceder’s group conducted the theoretical calculation to investigate the structural stability of highly desodiated NaMnCr(PO_4_)_3_, which suggests that Cr^4+^ migration is unlikely because both migration barrier and formation energy of Cr/Na antisites are large enough.^97^ They also excluded the Mn^3+^ Jahn–Teller effect as the possible reason for poor performance, as the combined analysis of XANES and EXAFS characterization suggests the structural distortion can be reversibly restored during cycling. By lowering the upper cutoff voltage (≤4.3 V) to shield the Cr^3+^/Cr^4+^ reaction, the reversible redox reactions of Mn^2+^/Mn^3+^ and Mn^3+^/Mn^4+^ can be achieved in the Na_4_MnCr(PO_4_)_3_ electrode; however, some capacity contributed from the low-voltage region and slight voltage hysteresis could be still observed from the voltage-capacity profiles.^100^ Therefore, it could be persuaded that the intrinsic structure defects related to Mn may also exist in Na_4_MnCr(PO_4_)_3_ in the as-prepared state. Further metal doping, stabilizer coating, and electrolyte optimization should be the main development direction of Na_4_MnCr(PO_4_)_3_ cathodes to reach the practical application level in future.

#### Mn–Zr/Al Compounds

The phase-pure Na_3_MnZr(PO_4_)_3_ was originally prepared by Hu’s group through the solid-phase method to serve as a cathode for NIBs.^1^ However, Na_3_MnZr(PO_4_)_3_ is nearly electrochemically inactive. The failure to extract Na^+^ reversibly could be the result of a solid-state synthesis that gives micrometer-sized crystallites and ineffective carbon coating. Considering this, Goodenough’s group used a sol–gel route to synthesize 200 nm level Na_3_MnZr(PO_4_)_3_ with an *in situ* thin carbon layer.^101^ With these particles, the reversible redox reactions of Mn^2+^/Mn^3+^ and Mn^3+^/Mn^4+^ could be achieved to allow a high discharge capacity of 105 mA h g^–1^ (Figure 5H) and 91% capacity retention at 0.5 C. By the combined theoretical calculation and experimental observations, their results indicate that the unfavorable Jahn–Teller distortion and disproportionation of Mn^3+^ could be effectively inhibited due to the synergistic effect of Mn and Zr (Figure 5I). More recently, Shen et al. applied a scalable spray-drying strategy to prepare Na_3_MnZr(PO_4_)_3_ microspheres with dual-carbon coating, which delivers decent electrochemical performance, especially low-temperature performance.^102^ Compared with Na_3_MnTi(PO_4_)_3_ analogues, Na_3_MnZr(PO_4_)_3_ allows lower theoretical capacity (based on two-electron reaction through Mn^2+^/Mn^3+^ and Mn^3+^/Mn^4+^) and higher cost of raw materials; therefore, Na_3_MnZr(PO_4_)_3_ may not be as promising as it sounds from an application perspective. In search of other suitable Mn-based cathodes, NASICON-type (space group of *R*3_2_) Na_4_MnAl(PO_4_)_3_ was considered an ideal candidate due to its low cost and high theoretical energy density.^103^ However, the poor electrochemical performance of NIBs limited their further development. From the aspect of composition design, some compositions such as Na_4_MnFe(III)(PO_4_)_3_ and Na_4_MnY(PO_4_)_3_ should be potential candidate cathodes; however, our results indicated that these compounds neither obtain pure phase nor achieve reversible Na insertion/extraction from the host.

### 4.2 Other Manganese-Based Polyanions

Manganese based compounds are generally derived from the iron based and vanadium-based analogues. Due to the advantages of high abundance, high voltage, and access to multielectron transfer (Mn^2+^/Mn^3+^/Mn^4+^), some manganese based polyanionic compounds with potential applications have been developed. Among them, the 3.8 V-level manganese-based mixed-phosphate cathode, i.e., Na_4_Mn_3_(PO_4_)_2_(P_2_O_7_), delivered significant potential for practical large-scale NIBs (Figure 5J).^104^ Na_4_Mn_3_(PO_4_)_2_(P_2_O_7_) is isostructural with orthorhombic Na_4_Fe_3_(PO_4_)_2_(P_2_O_7_), but there are some differences in the structural evolution during the charging/discharging process. Different from the solid-solution reaction of Na_4_Fe_3_(PO_4_)_2_(P_2_O_7_), Na_4_Mn_3_(PO_4_)_2_(P_2_O_7_) experienced several multiphase reactions (α, β, γ, and δ phase) based on Mn^2+^/Mn^3+^ redox couples. The total volume contraction/expansion of the electrode was calculated as ∼7%, which is slightly larger than that of Na_4_Fe_3_(PO_4_)_2_(P_2_O_7_) (∼4%) but smaller than most of the reported manganese-based cathodes, e.g., ∼ 10% of LiMnPO_4_. Interestingly, the first-principles calculations reveal that the Jahn–Teller distortion in Na_4_Mn_3_(PO_4_)_2_(P_2_O_7_) could facilitate sodium deinsertion/insertion kinetics due to opening up Na diffusion channels. As a result, the Na_4_Mn_3_(PO_4_)_2_(P_2_O_7_) electrode showed a high energy density of ∼416 W h kg^–1^ and reasonable high-temperature (60 °C) cycling performance. Mn dissolution in the electrolyte is another issue to affect the electrochemical performance of manganese-based cathodes. When robust artificial interphases between solid cathode and liquid electrolyte were designed, the cycling performance of Na_4_Mn_2_Fe(PO_4_)_2_P_2_O_7_ was significantly improved.^105^ Other manganese based polyanionic compounds, such as Na_3_MnPO_4_CO_3_,^106^ Na_2_MnSiO_4_,^107^ and triphylite-phase NaMnPO_4_,^108^ also rendered a high energy density over 350 W h kg^–1^; however, the sluggish kinetics and poor cycling stability make them less attractive as potential candidates for practical NIBs.

## 5 Conclusions and Outlook

**Figure 6:**
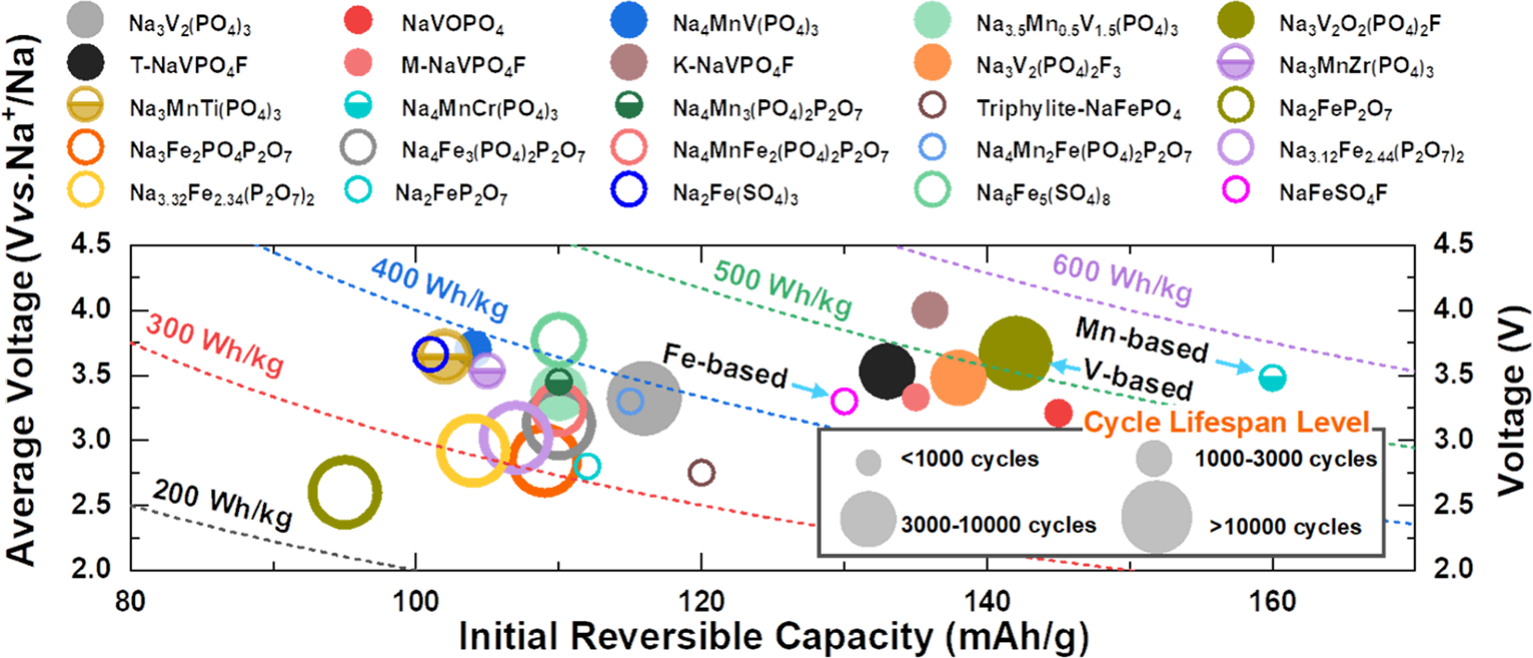
Summary of the reported electrochemical characteristics of typical polyanionic cathodes for NIBs, including average voltage, reversible capacity, energy density, and cycling lifespan. The energy density is calculated by integrating the area of discharge profile in half cells from the literature. The average voltage is obtained by dividing the energy density by the reversible capacity. The cycle life was defined as the cycle number based on 70% retention of the initial capacity. KTiOPO_4_-type, monoclinic, and tetragonal NaVPO_4_F were denoted as K-NaVPO_4_F, M-NaVPO_4_F, and T-NaVPO_4_F, respectively.

The polyanionic cathodes for NIBs have attracted wide attention due to their stable structure, high voltage output, and good safety in recent years. However, the current polyanionic compounds are still a way from being commercially competitive cathodes for NIBs. As is well known, to improve the electronic/ionic kinetics, the typical polyanionic cathodes should possess nanoscale particle sizes and a necessary carbon coating; however, these lead to a relatively low volume energy density, which is an obstacle for the practical use of NIBs. In this sense, development of an advanced synthesis route to prepare large-sized and dense particles of polyanionic cathodes is an imperative. The enhanced electronic structure-by-structure design (e.g., composition optimization, cation/anion doping) was also expected to improve the intrinsic electron conductivity of polyanionic cathodes, thus avoiding the introduction of excess conductive carbon into cathode or electrode levels. Structural optimization and interface design are important factors in maintaining polyanionic cathode cycling stability. Simultaneously achieving satisfactory energy density and long cycling life is critical and challenging for the current polyanionic cathodes to serve as large-scale stationary energy storage devices. Here, the key parameters (i.e., reversible capacity, voltage output, and cycle lifespan) of the representative polyanion-type cathodes (including V-, Fe-, and Mn-based compounds) for NIBs from the reported literature are summarized in Figure 6. Despite some exciting breakthroughs having been achieved, further improvement of the comprehensive performance of these composites is still currently required.

The mixed vanadium-based phosphates (mainly sodium vanadium fluorophosphate) are concentrated in the high energy density region of ∼500 W h kg^–1^, and most of them show a desirable cycling lifespan of over 3000 cycles, which distinguishes them from other polyanionic compounds. However, compared to the iron- and manganese-based compounds, the vanadium-based cathodes deliver higher raw material cost, which weakens their advantages. Moreover, the solution-based synthesis methods are usually accompanied by nanoscale morphology and small particle sizes, thereby lowering the compacted density and practical energy density. Slowing down the nucleation and growth rate through the introduction of a proper chelating agent and optimization of relevant synthesis parameters to obtain dense spherical particles with large size could be an alternative route to further increase the practical energy density.

Iron-based polyanion compounds usually showed a desirable cycling lifespan but inferior energy density due to the relatively low potential rendered by Fe^2+^/Fe^3+^ redox couples. By introducing anions with strong electronegativity, e.g., P_2_O_7_^4–^, F^–^, and SO_4_^2–^, the corresponding voltage output increased significantly. Especially, the SO_4_^2–^-rich Na_2+2*x*_Fe_2–*x*_(SO_4_)_3_ family demonstrated over 3.6 V-level average voltage and outstanding energy density that was close, and even superior, to 400 W h kg^–1^ as well as notably low raw material costs, which makes them become one of the “star” cathodes for NIBs. However, nonstoichiometric impurity and a fatal humidity sensitivity greatly hindered their practical application. Development of an effective solution-based or other advanced synthesis route to promote the phase purity of Na_2+2*x*_Fe_2–*x*_(SO_4_)_3_ is urgent and imperative. Besides, the real structural origin for the strong hygroscopicity still remains elusive so far. Alternatively, effective construction of a hydrophobic particle surface by dense carbon coating or extensionally growing a stable isomorphic layer could be expected to improve the air stability and electrochemical performance of the Na_2+2*x*_Fe_2–*x*_(SO_4_)_3_ family.

Manganese based polyanionics are extremely attractive for NIBs on account of their high abundance and available two-electron redox reaction of the Mn^2+^/Mn^3+^/Mn^4+^ couples. Despite V^3+^/V^4+^or V^4+^/V^5+^ redox couples having been also demonstrated in polyanion compounds for NIBs, in most cases, fully and simultaneously utilizing the reactions of V^3+^/V^4+^/V^5+^ couples have not been achieved due to structural degradation related to V^5+^. Based on this point, manganese combining with another active element in the polyanion enables a three-electron redox reaction and thereby increases energy density. Theoretically, Na_4_MnV(PO_4_)_3_ is an ideal candidate cathode for reaching a large capacity of over 150 mA h g^–1^ through access to V^3+^/V^4+^, Mn^2+^/Mn^3+^, and Mn^3+^/Mn^4+^ redox couples in the proper voltage window of 2.5–4.2 V. Unfortunately, the irreversible V^4+^/V^5+^ reaction takes precedence over the Mn^3+^/Mn^4+^ oxidation, finally leading to a failure to reach the reversible three electron reaction. It will be of great significance to improve the reversibility of V^4+^/V^5+^ or avoid/delay the occurrence of V^4+^/V^5+^ in Na_4_MnV(PO_4_)_3_ from structure design aspects. Another dazzling manganese based cathode is Na_4_MnCr(PO_4_)_3_, which has revealed a large reversible capacity of over 150 mA h g^–1^ by utilizing Mn^2+^/Mn^3+^/Mn^4+^ and Cr^3+^/Cr^4+^ redox couples. As discussed above, the high off-cut voltage (∼4.6 V) and poor cycling performance have been huge obstacles for the application of Na_4_MnCr(PO_4_)_3_. Future endeavors are welcomed in electrolyte optimization and interfacial engineering, which could push Na_4_MnCr(PO_4_)_3_ to real application situations.
